# The impact of using reinforcement learning to personalize communication on medication adherence: findings from the REINFORCE trial

**DOI:** 10.1038/s41746-024-01028-5

**Published:** 2024-02-19

**Authors:** Julie C. Lauffenburger, Elad Yom-Tov, Punam A. Keller, Marie E. McDonnell, Katherine L. Crum, Gauri Bhatkhande, Ellen S. Sears, Kaitlin Hanken, Lily G. Bessette, Constance P. Fontanet, Nancy Haff, Seanna Vine, Niteesh K. Choudhry

**Affiliations:** 1 Center for Healthcare Delivery Sciences, Division of Pharmacoepidemiology and Pharmacoeconomics, Department of Medicine, Brigham and Women’s Hospital and Harvard Medical School, Boston, MA USA; 2 Microsoft Research, Herzliya, Israel; 3 Tuck School of Business, Dartmouth College, Hanover, NH USA; 4 Division of Endocrinology, Diabetes and Hypertension, Department of Medicine, Brigham and Women’s Hospital and Harvard Medical School, Boston, MA USA

**Keywords:** Health services, Outcomes research, Public health, Type 2 diabetes, Drug therapy

## Abstract

Text messaging can promote healthy behaviors, like adherence to medication, yet its effectiveness remains modest, in part because message content is rarely personalized. Reinforcement learning has been used in consumer technology to personalize content but with limited application in healthcare. We tested a reinforcement learning program that identifies individual responsiveness (“adherence”) to text message content and personalizes messaging accordingly. We randomized 60 individuals with diabetes and glycated hemoglobin A1c [HbA1c] ≥ 7.5% to reinforcement learning intervention or control (no messages). Both arms received electronic pill bottles to measure adherence. The intervention improved absolute adjusted adherence by 13.6% (95%CI: 1.7%–27.1%) versus control and was more effective in patients with HbA1c 7.5- < 9.0% (36.6%, 95%CI: 25.1%–48.2%, interaction *p* < 0.001). We also explored whether individual patient characteristics were associated with differential response to tested behavioral factors and unique clusters of responsiveness. Reinforcement learning may be a promising approach to improve adherence and personalize communication at scale.

## Introduction

Text messages can be delivered at low cost and provide reminders, education, and motivational support for health behaviors on an ongoing basis^1^. They have demonstrated effectiveness for supporting physical activity, medication adherence, and other daily self-management activities that are guideline recommended for managing chronic diseases, like type 2 diabetes^2,3^. However, many prior text messaging interventions have used generic message content (i.e., the same messages delivered to all patients)^4–6^.

Yet, a key principle for changing health behaviors is personalization and how information is presented to match an individual’s specific needs, which may also change over time^7–10^. Personalization can be based upon simple characteristics, such as name, age, or health metrics^11^. More detailed personalization could potentially be achieved by incorporating routines or behavioral barriers, and adjusting frequently^12,13^.

A major obstacle to achieving personalization based on underlying behavioral tendencies is the ability to predict what patients will actually respond to. Traditionally, theory-based assessments, or expert opinion (like barrier elicitation by clinicians), have been used to tailor behavioral messaging particularly at the outset, and sometimes, with updates at intervals^14–18^. For example, the REACH trial used interactive texts that asked participants directly about their adherence through weekly feedback, and another recent trial used dynamic tailoring based on patients’ implementation intention plan^17,18^. An alternative approach is to use observations of what content patients actually respond to and use that as the basis for what they will respond to in the future. This process is made feasible by mobile health tools (like electronic pill bottles) that passively measure health behaviors on an ongoing basis. Consistent with this, there is emerging interest in just-in-time adaptive interventions (JITAIs), or an intervention design that adapts support (e.g., type, timing, intensity) over time in response to an individual’s changing status and context^19,20^.

An efficient approach to achieve such personalized intervention is with the use of reinforcement learning^21,22^. This machine learning method trains a statistical model based on rewards from actions of the model in an environment. In the context of behavior change, the model observes individual behaviors in response to cues it provides (like text messages) and learns to optimize response (like adherence) through systematic trial-and-error^23,24^. This technique has technological underpinnings applied in computer gaming and robotics^21,25–27^. In contrast to other approaches to achieving personalization, reinforcement learning uses approaches that predict the effectiveness of different intervention components and also can use latently derived estimates for tailoring (rather than end user input); and, as interventions are deployed, updates the predictions based on their successes and failures (both at the individual and group level)^28^. That is, the algorithm “learns” to personalize as it experiments, or “adapts”^29^.

Reinforcement learning has thus far had limited use in health care^27,28,30–32^ and has not been applied to medication adherence, an essential daily activity for most patients with chronic disease, and especially diabetes, which affects 529 million individuals globally^2,33^. While machine learning generally has been shown to be helpful in measuring suboptimal adherence^34,35^, there remains much opportunity to explore how it and related techniques can improve adherence. Accordingly, we launched the REinforcement learning to Improve Non-adherence For diabetes treatments by Optimizing Response and Customizing Engagement trial (REINFORCE) to evaluate the impact of a text messaging program tailored using reinforcement learning on medication adherence for patients with type 2 diabetes^22^.

The trial design has been published^22^ with expanded details in the Methods. In brief, 60 patients with type 2 diabetes (with their latest glycated hemoglobin A1c [HbA1c] lab value ≥ 7.5% in the past 180 days) were randomized to a reinforcement learning intervention or control (no intervention) based on pre-specified power calculations. In both arms, patients received a separate electronic pill bottle for each of their diabetes medications, with bottles that look like those dispensed by retail pharmacies but with an electronic cap that recorded the dates and times in which participants took their medications. A figure of the infrastructure was previously published^22^. The reinforcement learning algorithm personalized daily texts based on adherence, patient characteristics, and message history using the following 5 behavioral factors: (1) how the messages are structured (“Framing”; classified as neutral, positive [invoking positive outcomes of medication use], or negative [invoking consequences of medication non-use]), (2) observed feedback (“History”, i.e., including the number of days in the prior week the patient was adherent), (3) social reinforcement (“Social”, i.e., referring to loved ones), (4) whether content was a reminder or informational (“Content”), and (5) whether the text included a reflective question (“Reflective”). Individual messages contained elements from these different factor sets, examples of which have been published previously^22^. The primary outcome was average pill bottle-measured adherence over a 6-month follow-up. After trial completion, we described the performance of the reinforcement learning algorithm process itself and explored responsiveness to behavioral factors using subgroup analyses and clustering methods, as prior work has suggested that there may be important differences in responsiveness^24^.

## Results

**Fig. 1:**
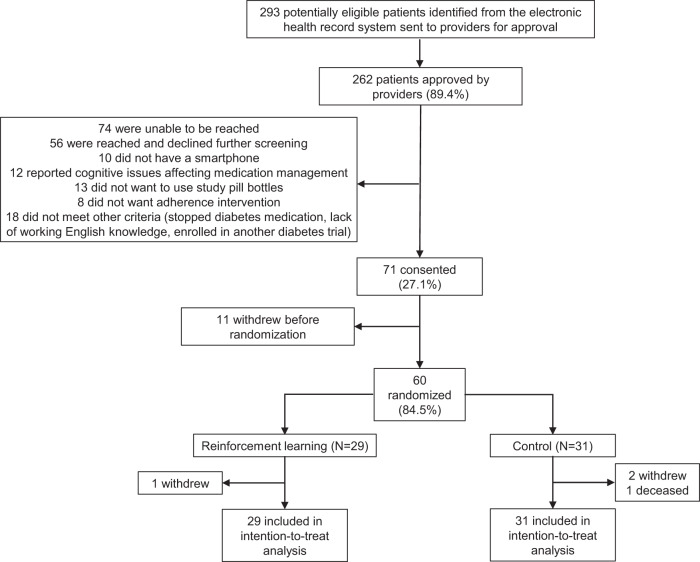
CONSORT diagram. This diagram shows a visual representation of the flow of patients through the trial.

Among 60 patients, 29 and 31 were randomized to the intervention and control arms, respectively, of which 1 intervention and 3 control patients did not complete follow-up (Fig. 1). All 60 patients were included in the intention-to-treat analysis.

In total, 26 patients (43%) were female and 35 (58%) were White (Table 1). Baseline characteristics were slightly different between the arms based on absolute standardized differences but were well-balanced on key metrics including age, sex, baseline HbA1c values, and baseline adherence. Intervention group patients had less formal education (e.g., 24.1% vs. 16.1% having no more than a high school education) and took more oral diabetes medications (31% vs. 19% taking ≥2 medications) versus control patients.

**Table 1:** Baseline characteristics of trial patients

	Reinforcement learning (*n* = 29)	Control (*n* = 31)	Absolute standardized differences
Age, mean (SD)	60.3 (11.4)	56.7 (12.9)	0.02
Female sex, *n* (%)	12 (41.4%)	14 (45.2%)	0.08
Race/ethnicity, *n* (%)			
White	17 (58.6%)	18 (58.1%)	0.01
Black or African American	6 (20.7%)	8 (25.8%)	0.12
Other (Hispanic/Latino, Asian, Other)	6 (20.7%)	7 (22.6%)	0.05
Education level, *n* (%)			
High school graduate or below	7 (24.1%)	5 (16.1%)	0.20
Some college/college graduate	22 (51.7%)	19 (61.3%)	0.19
Post-graduate	7 (24.1%)	7 (22.6%)	0.04
Married or partnered, *n* (%)	13 (44.8%)	17 (54.8%)	0.20
Baseline HbA1c, mean (SD)	8.99 (1.39)	9.10 (1.47)	0.06
<9, *n* (%)	17 (58.6%)	17 (54.8%)	0.08
≥9, *n* (%)	12 (41.4%)	14 (45.2%)	0.08
Diabetes medication use (self-reported), n (%)			
<4 years	10 (34.5%)	13 (41.9%)	0.15
≥4 years	19 (65.5%)	18 (58.1%)	0.15
3 or more unique physicians, *n* (%)	19 (65.5%)	16 (51.6%)	0.29
Number of medications, *n* (%)			
1	20 (69.0%)	25 (80.6%)	0.27
≥2	9 (31.0%)	6 (19.4%)	0.27
Non-adherence (number of self-reported doses missed in prior 30 days), %			
≤1	17 (58.6%)	17 (54.8%)	0.08
>1	12 (41.4%)	14 (45.2%)	0.08
Automaticity^a^ with medication-taking, *n* (%)	5 (17.2%)	10 (32.3%)	0.36
Full patient health activation^b^	16 (55.2%)	15 (48.4%)	0.13

This table shows the baseline characteristics of the 60 randomized patients to the trial.

*SD* Standard deviation, *HbA1* glycated hemoglobin A1c.

^a^Highest possible score based on the automaticity subscale of the self-report behavioral automaticity index.

^b^Full patient activation based on the consumer health activation index.

### Description of the reinforcement learning algorithm learning process

In total, 5143 text messages were sent to patients in the intervention arm (*n* = 29) during the 6-month study period. Intervention patients received daily messages; an average of 27.7 (SD: 5.9) unique messages were sent to each patient (Table 2). In aggregate, 514 (10.0%), 2473 (48.1%), and 2058 (40.0%) of text messages contained ≥3, ≥4, and ≥5 behavioral factors respectively.

**Table 2:** Adherence as a function of the behavioral factors included in the text messages among intervention patients

	Framing (Positive)	Framing (Negative)	History (Observed feedback)	Social	Content	Reflective
Adherence on the day the factor was included in the text message^a^, mean (SD)	76.1 (38.4)	81.1 (34.6)	90.7 (23.8)	76.1 (38.4)	76.3 (38.2)	76.2 (38.3)
Adherence on the day after the factor was included in the text message^a^, mean (SD)	75.8 (38.7)	81.9 (33.4)	90.2 (24.8)	75.7 (38.7)	76.0 (38.5)	76.1 (38.5)

This table shows the average adherence on the day and the day after a text message with these factors was selected and sent to intervention arm patients.

*SD* Standard deviation.

^a^Average adherence is measured by pill bottles in the day of interest and averaged across patients.

The reinforcement learning algorithm also adapted its selection of behavioral factors in the text messages; the proportions of intervention arm patients who received the five factors over the trial are shown descriptively in Supplemental Fig. 1 panels. For example, positive framing as a factor (Supplemental Fig. 1a) was initially not frequently selected by the algorithm during the first two months of the trial but became more prevalent later. By contrast, negative framing was more commonly selected at first but decreased over time (Supplemental Fig. 1b). Other plots for receipt of history, social reinforcement, content, and reflection are shown in Supplemental Fig. 1c–f, respectively. More patients were selected to receive social reinforcement, content, and reflection as factors as the trial progressed, while the proportion of patients receiving history (observed feedback) remained relatively equal over time.

**Fig. 2:**
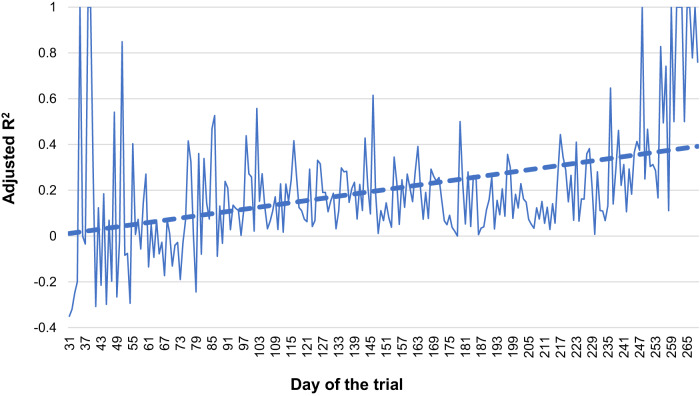
Change in reinforcement learning algorithm adjusted R^2^ over the course of the trial. The adjusted R^2^ from trial calendar day 31 to 279 (March 13, 2021–December 19, 2021) is plotted from each day’s model. We calculated adjusted R^2^ from the proportion of variance in daily adherence that is explained by the five intervention factors in the reinforcement learning model. We selected those windows as they each had a minimum of 5 patient observations that day.

Figure 2 shows the change in adjusted R^2^ of the reinforcement learning algorithm over the trial. This statistic, which describes the extent to which adherence is explained by algorithm predictions for behavior following a message sent to participants, increased over time, indicating that the algorithm learned to send more effective messages to patients.

**Fig. 3:**
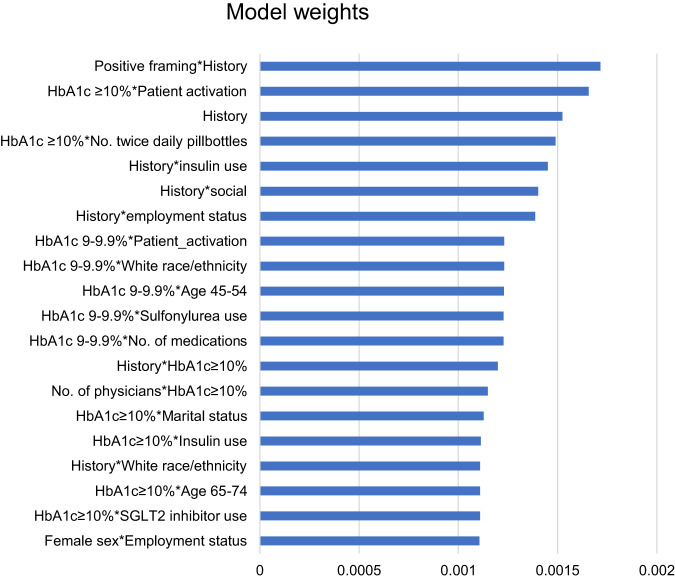
Most influential feature interactions in the reinforcement learning algorithm. This figure shows the model weights from the feature importance score from the reinforcement learning algorithm, which indicates which features were more or less importance to the model. The weights above were the 20 most influential features, ranked from highest to lowest. Abbreviations: HbA1c, glycated hemoglobin A1c; SGLT2, sodium-glucose cotransporter-2.

The most influential features and interactions from the reinforcement learning algorithm are shown in Fig. 3. Fixed characteristics that carried the most weight within the model were baseline HbA1c, self-reported level of patient activation, number of medications included in electronic pill bottles, concomitant insulin use, and employment status based on their interactions. The behavioral factors with the largest weight included positive framing, observed feedback, and social reinforcement.

### Effect of the reinforcement learning intervention on the primary outcome

**Fig. 4:**
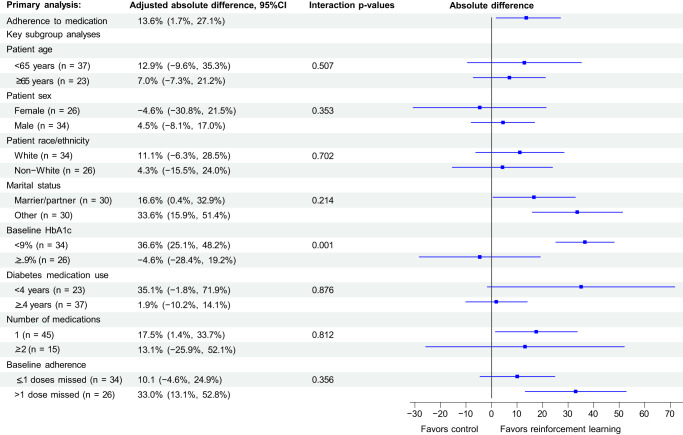
Trial primary outcome and subgroup analyses. We used generalized estimating equations with an identity link and normally-distributed errors to evaluate the effect of the intervention on adherence to medication measured by pill bottles compared with control. The points on the figure are the point estimates and the error bars are the 95% confidence intervals from the relevant sample sizes. These models were adjusted for baseline characteristics and the block randomized design. The primary outcome is shown at the top. The results of exploratory subgroup analyses by key demographic and clinical characteristics also shown; these were performed by repeating the same models within each subgroup, using interaction p-values to assess between subgroups.

Over the 6-month follow-up, average adherence to medication was 74.3% (SD: 30.8%) in the reinforcement learning intervention arm compared with 67.7% (SD: 29.4%) in the control arm (Fig. 4). After adjusting for the block randomized design and baseline characteristics, average adherence among intervention patients was 13.6% (95%CI: 1.7%, 27.1%, *p* = 0.047) higher than control (shown in Fig. 4). Sensitivity analyses, including omitting the first two weeks of pill bottle data and censoring patients in both arms after 30 days of pill bottle non-use (3 patient and 1 patient in intervention and control arms, respectively) did not change the results (Supplemental Table 1).

Hypothesis-generating demographic and clinical subgroup analyses that explored interactions between patient characteristics and the intervention’s effectiveness on adherence are also shown in Fig. 4. The strongest interaction between the overall effectiveness of reinforcement learning and adherence was by baseline HbA1c level. Specifically, in patients with HbA1c 7.5- < 9.0%, the intervention improved adherence by 33.6% (95%CI: 15.9%, 51.4%) versus control contrasted with those with baseline HbA1c ≥ 9% (interaction *p* value: 0.001) in which there was no significant difference compared with control. In patients who were non-adherent at baseline (i.e., self-reported missing >1 medication dose in the 30 days before enrollment), the intervention improved adherence by 33.0% (95%CI: 13.1%, 52.8%) versus control, but this interaction was not significant (interaction *p* value: 0.214).

### Exploratory analyses of responsiveness to behavioral factors

**Fig. 5:**
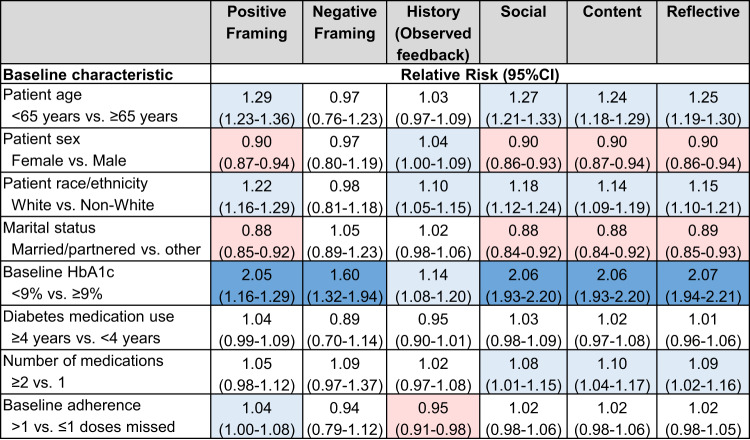
Relationship between patient characteristics and responsiveness to individual behavioral factors. This figure shows the results of these exploratory analyses with the outcome being optimal adherence (adherence=1) for the day after the factor was selected and sent within the text message. We used generalized estimating equations for each behavioral factor with a log link and binary-distributed errors, adjusted for patient baseline characteristics but unadjusted for patient-level clustering. Light red indicates a negative association (Relative risk 0.50–0.99); Light blue indicates a positive association (Relative risk 1.01–1.50); and Dark blue indicates a strong positive association (Relative risk ≥1.50). Abbreviations: CI Confidence interval, HbA1c glycated hemoglobin A1c.

In hypothesis-generating analyses, we whether responsiveness to the tested behavioral factors (determined by optimal adherence) differed by patient baseline characteristics. As shown in Fig. 5, patients who were aged <65 years (compared with ≥65), were of White race/ethnicity (compared with non-White), had HbA1c < 9% (compared with ≥9%), were of other marital status (compared with married/partnered), and were taking multiple medications (compared with 1) responded better than their counterparts to most or almost all behavioral factors. In contrast, women were more responsive to messages reporting their medication-taking history than men but were less responsive to other factors. Finally, patients who were more non-adherent at baseline (self-reported missing >1 dose, compared to those who reported missing ≤1 doses in the last 30 days) were more responsive to positively-framed messages and less responsive to messages reporting their medication-taking history, but had similar responsiveness to all other factors.

**Fig. 6:**
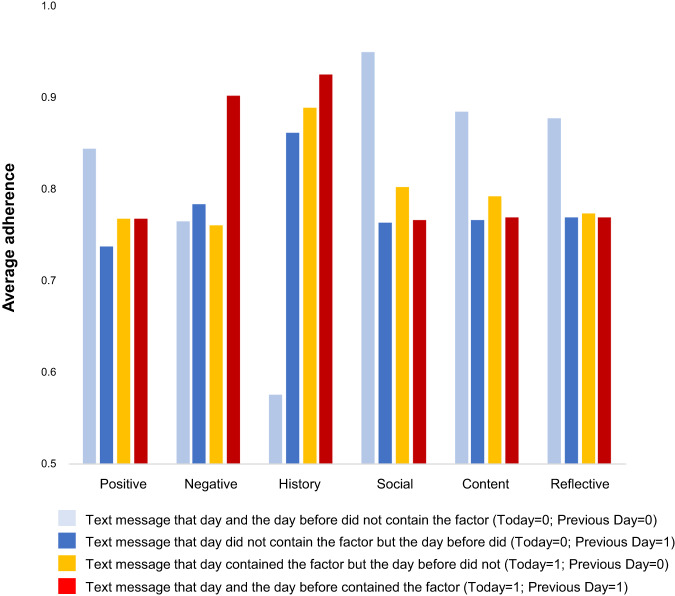
Average adherence across behavioral factors stratified by the sequence of text messages the prior day and the same day the message was sent. This figure shows the average daily adherence measured by pill bottle over the course of the trial among the 29 intervention arm participants. These results are stratified by the text message sent in the prior day and/or the same day contained that intervention factor (e.g., positive framing). For example, the dark blue bar for “positive framing” indicates the level of adherence if the prior’s day text message contained positive framing but that day’s text message did not.

Adherence differed based on whether that behavioral factor had been sent the prior day (Fig. 6). For instance, adherence was highest when negatively-framed messages and messages containing observed medication feedback were sent two days in a row (i.e., red columns). By contrast, no difference in adherence was observed when text messages including and not including the behavioral factor were alternated.

**Fig. 7:**
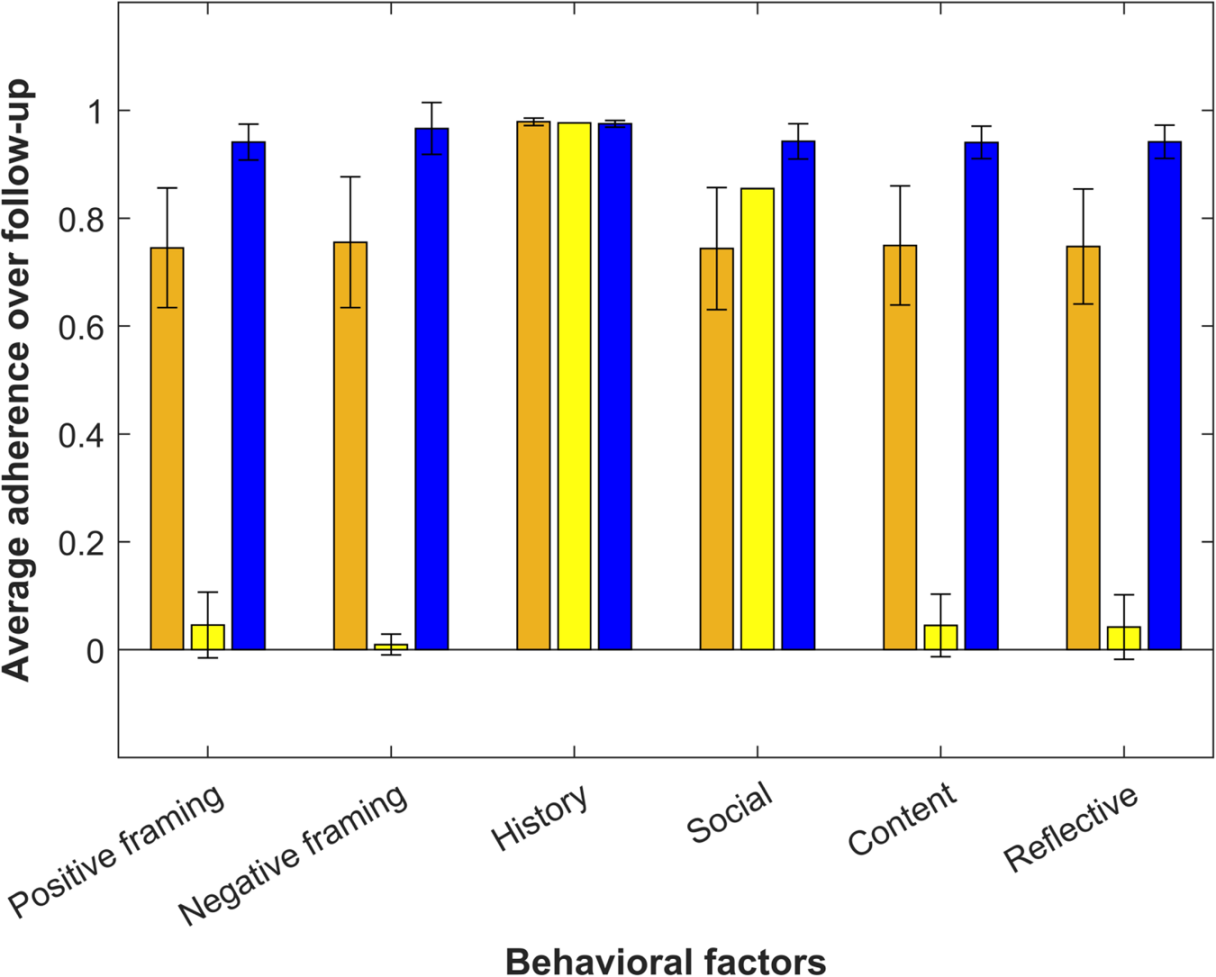
Clusters of responsiveness to behavioral factors included in text messages. Each color represents one of the three different patient groups identified from the exploratory *k*-means clustering analysis for the average pill bottle adherence measured over 6 months (primary outcome). These groups include: (1) Group 1 (Orange, *n* = 9) was the most adherent in response to observed feedback (“history”), (2) Group 2 (Yellow, *n* = 4) was the most adherent in response to social reinforcement or observed feedback, and (3) Group 3 (Blue, *n* = 16) was equally adherent in response to all types of messages. The error bars show the standard error for the cluster based on the underlying sample size (i.e., a threshold of ≥25 observations was applied).

Using *k-*means clustering analysis of average adherence given the behavioral factors, we identified three unique patient clusters (Fig. 7). These clusters included: (1) Group 1 (Orange, *n* = 9) responding best to observed feedback, (2) Group 2 (Yellow, *n* = 4) responding best to social reinforcement and observed feedback, and (3) Group 3 (Blue, *n* = 16) responding equally to all message types. Individuals who were married/partnered were more likely to be in Group 1 compared with the other two groups, but most associations were non-significant owing at least in part to small sample size (Table 3).

**Table 3:** Relationship between baseline characteristics and membership in clusters of responsiveness to behavioral factors

Referent: Group 3 Baseline Characteristic	Odds Ratio (95% CI) for Group 1	Odds Ratio (95% CI) for Group 2
Age: ≥65 years vs. <65 years	0.80 (0.16–4.12)	0.33 (0.03–3.92)
Sex: Female vs. Male	1.33 (0.25–7.01)	1.67 (0.18–15.13)
Race/ethnicity: White vs. Non-White	0.23 (0.04–1.30)	1.36 (0.11–16.58)
Partner status: Married/partnered vs. other	7.70 (1.15–51.15)^a^	0.73 (0.06–8.92)
Diabetes control: baseline HbA1c < 9% vs. ≥9%	0.19 (0.03–1.14)	Too small to compute
Diabetes medication use: ≥4 years vs. <4 years	4.80 (0.48–48.46)	0.20 (0.02–2.39)
Number of medications: 1 vs. ≥2	2.10 (0.32–13.61)	1.80 (0.15–21.48)
Self-reported baseline adherence: >1 dose missed vs. ≤1 dose missed	0.64 (0.12–3.53)	1.29 (0.14–11.54)

Among 29 intervention arm patients, we used exploratory multinomial logistic regression comparing baseline patient characteristics of those in Group 1 (responding best to observed feedback [history], *n* = 9, Orange in Fig. 6) and Group 2 (responding best to social reinforcement or observed feedback, *n* = 4, Yellow in Fig. 6) using Group 3 as the referent group (responding equally to each factor, *n* = 16, Blue in Fig. 6).

^a^*p* < 0.05.

*CI* Confidence Interval.

## Discussion

In this randomized-controlled trial of a reinforcement learning intervention that personalized text messaging content for patients with diabetes (and HbA1c ≥ 7.5%, above most guideline targets), we found that the intervention improved adherence to medication over a 6-month follow-up. The intervention was particularly effective among patients with HbA1c between 7.5 and 9.0%. Adherence changes of this magnitude have been associated with differences in patient outcomes and health care spending^36,37^.

Numerous trials have demonstrated that text messages support adherence to medication^1,4–6,11,38–40^. However, the effectiveness of many prior approaches has been limited, in part because they have not personalized the content and presentation of the messages patients receive^38^. To our knowledge, no study has personalized text messages for adherence in real-time on a daily basis through latent measurement of adherence and response, especially using reinforcement learning. Some prior work has personalized text messages for adherence based on simple user characteristics, preferences or self-reported adherence, at pre-specified intervals, or through relatively static “if-then” rules, but have not adapted based on observing what patients respond to^11,17–19^. Reinforcement learning has indicated early promise for other health behaviors. For example, a 3-arm trial of 27 patients tested the impact on physical activity of different text messaging approaches for individuals with type 2 diabetes, finding that text messaging using reinforcement learning resulted in significant more physical activity and lower HbA1c values than non-personalized weekly texting strategies^24,27^. Reinforcement learning interventions for titrating anti-epilepsy medications and selecting sepsis protocols have also demonstrated effectiveness^31,32,41^.

The reinforcement learning intervention appears to have learned from patient observations and changed the messages that it selected over time. This was particularly evident in its approach to message framing. The algorithm initially favored negatively framed messages (e.g., highlighting the negative disease consequences of non-adherence to medication) but over time, there was a noticeable shift such that more patients received either a neutral tone or positively framed message (e.g., highlighting positive consequences of adherence). This change was also seen quantitatively with the increasing proportion of variance in daily adherence explained by the behavioral factors in the text messages (i.e., the adjusted R^2^). By the end of the trial, the adjusted R^2^ was consistently over 0.40, meaning that much of the difference in adherence on a given day could be explained by the five algorithm factors. Additional features, for example, the interaction between positive framing and observed feedback as well as higher HbA1c and patient activation provided the greatest weight to the model prediction, suggesting that the algorithm incorporated learned information. Together, these findings suggest that the reinforcement learning algorithm not only changed its strategy over time but also improved its performance in predicting what types of messages would improve individuals’ adherence.

The intervention was also particularly effective in patients with HbA1c between 7.5 and 9.0%. The reason for this can be explained in two ways; first, patients further from guideline targets may need treatment intensification in addition to better adherence to their existing medications^2,3^. Second, a prior trial also suggested that individuals have varying preferences for how to escalate diabetes care at different levels of HbA1c values; those with HbA1c between 7.5 and 9.0% were more interested in adherence support than other interventions^16^. While less pronounced and not statistically significant, patients reporting worse adherence at baseline also tended to respond more to the intervention. This may also have been due to the fact that individuals who report missing multiple doses in the last 30 days most likely have substantial non-adherence^42^ and are an ideal target population for an adherence intervention.

Supporting the potential benefits of personalization and for generating future hypotheses, we explored characteristics of patients who responded differently to different message types. The most notable was that women responded better to receiving observed feedback about their medication-taking than men but responded less well to positive framing, social messaging, informational messaging rather than reminders, and messages that were intended to provoke reflection. One explanation could be that some women are already aware of how their own health can benefit loved ones and may prefer more straightforward reminders and feedback about their medication-taking performance^24,43^, although future work should explore further within larger sample sizes.

In our exploratory analyses, there were clusters of patients who responded to different types of messages. In specific, one group responded best to observed feedback, and a second group responded best to social reinforcement and observed feedback, while a third responded equally to all types of messages. We also found higher adherence when negatively-framed messages and messages that contained observed feedback were provided two days in a row, perhaps reflecting the need to reinforce these types of messages, but not others. The fact that the algorithm de-prioritized negative framing on average over time but that it was effective in combination with observed feedback is also worthy of consideration. This could in part be explained by underlying heterogeneity of the patient population in their responsiveness, and in how the information was sequenced, emphasizing the potential impact of personalization but should be explored further.

Future work could extend these findings in several ways. First, researchers should test the added impact of using reinforcement learning with non-personalized text messages. Second, the impact of a reinforcement learning intervention should be tested on long-term clinical outcomes and in a larger and more diverse sample to confirm some of the exploratory analyses about responsiveness to different behavioral factors. Finally, this work could be applied in other ways, for example to other disease states or related guideline-recommended daily activities such as physical activity or diet.

Several limitations should be acknowledged. First, electronic pill bottles could have influenced adherence, especially during the initial period of observation; however, they have been shown to correlate strongly with actual pill consumption^44,45^, and we minimized this observer bias by using pill bottles in both arms. While we powered the study to detect a 10% difference in adherence, the standard deviations were wider than anticipated, likely owing to the small sample size and overall heterogeneity in medication-taking than previously observed^46^. The findings may also not generalize to patients with pre-diabetes or gestational diabetes or those without reliable access to a smartphone. The subgroup and responsiveness analyses were also limited by small sample sizes and should be considered exploratory. It is also currently technologically less feasible to passively measure adherence to injectable agents in a scalable manner, and oral diabetes medications are the cornerstone of first and second-line type 2 diabetes treatments. Finally, we also chose not to have a “generic” text messaging arm, in part to test the highest possible efficacy of the intervention so we cannot assess the incremental benefit of personalization versus generic messaging with this design.

In conclusion, the reinforcement learning intervention led to improvements in adherence to oral diabetes medication and was particularly effective in patients with HbA1c between 7.5 and 9.0%. This trial provides insight into how reinforcement learning could be adapted at scale to improve other self-management interventions and provides promising evidence for how it could be improved and tested in a wider population.

## Methods

### Study design

Trial design details have been previously published^22^. The protocol was designed, written, and executed by the investigators (Fig. 1). Study enrollment began in February 2021 and completed in July 2021. Follow-up of all patients ended in January 2022; the final study database was available in March 2022.

### Study population and randomization

The trial was conducted at Brigham and Women’s Hospital (BWH), a large academic medical center in Massachusetts, USA. Potentially-eligible patients were individuals 18–84 years of age diagnosed with type 2 diabetes and prescribed 1–3 daily oral diabetes medications, with their most recent glycated hemoglobin A1c (HbA1c) level ≥7.5% (i.e., above guideline targets)^47^. These criteria were assessed using BWH electronic health record (EHR) data. To be included, patients also had to have a smartphone with ability to receive text messages, have working knowledge of English, not be enrolled in another diabetes trial at BWH, not use a pillbox or switch to using electronic pill bottles for their diabetes medications for the study, and be independently responsible for taking medications. Smartphone connectivity was essential to measure daily adherence, but they have been widely adopted, even among patients from socioeconomically disadvantaged backgrounds^48,49^. Patients using insulin or other diabetes injectables in addition to their oral medication were allowed to be included to enhance generalizability.

As previously described^22^, potentially eligible patients with a recent or upcoming diabetes clinic visit were identified from the EHR on a biweekly basis. Once identified, the patients’ endocrinologists were contacted for permission to include their patient(s) in the study. Patients approved for enrollment were sent a letter on their endocrinologist’s behalf inviting them to participate and were then contacted by telephone. Patients who agreed provided their written informed consent captured through REDCap electronic data capture tools^50,51^, completed a baseline survey containing measures including demographics, self-reported adherence^42^, health activation^52^, and automaticity^53^ of medication-taking, and were mailed a separate Pillsy^®^ electronic pill bottle for each of their eligible diabetes medications (i.e., each patient received between 1–3 pill bottles). Electronic pill bottles have been widely used in prior research and have shown high concordance with other measurement methods^44,54^. The data from the pill bottles were transmitted through the patients’ smartphones via an app that otherwise had no features enabled for the app or pill bottles (i.e., any latent adherence reminders through the pill bottle were turned off). A figure of the infrastructure has been previously published^22^.

After receiving the pill bottles, patients were randomized in a 1:1 ratio to intervention or control using block randomization based on baseline level of self-reported adherence (i.e., ≤1 dose or >1 doses missed in the last 30 days^42^) and (2) baseline HbA1c of <9.0% or ≥9.0%^2^. Patients were asked to use these devices instead of regular pill bottles or pillboxes for their eligible oral diabetes medications. After randomization, patients were followed for 6 months for outcomes. At the end of follow-up, patients were contacted to complete a follow-up survey and ensure complete synchronization of their pill bottles. Both arms received a $50 gift card for participation.

### Intervention

The intervention was a reinforcement learning text messaging program that personalized daily text messages based on the electronic pill bottle data. Messages were selected by the Microsoft Personalizer^®^ algorithm^22,24^, a reinforcement learning system which aimed to achieve the highest possible sum of “rewards” over time, and which adapted over time by monitoring the success of each message to nudge patients to adhere to their medications.

The messages were based on behavioral science principles of how content influences patient behavior^55–57^. Based on qualitative interviews^58^, we selected 5 behavioral factors for the messages: (1) framing (classified as neutral, positive [invoking positive outcomes of medication use], or negative [invoking consequences of medication non-use]), (2) observed feedback (“History”, i.e., including the number of days in the prior week the patient was adherent), (3) social reinforcement (“Social”), (4) whether content was reminder or informational (“Content”), and (5) whether the text included a reflective question (“Reflective”)^7,8,24,59–61^. We designed ≥2 text messages for each unique set of factors (i.e., 47 unique sets across 128 text messages); examples of the factors sets contributing to the reinforcement learning model have been published^22^.

Every day, adherence from the prior day was measured by the electronic pill bottles, with values ranging from 0 to 1 based on the fraction of daily doses taken across their diabetes medications, averaging if they are taking multiple medications^22^. These served as the “reward” events used to provide feedback to Microsoft Personalizer^®^. The algorithm learned to predict which factors should have been included in the message on a given day to maximize the rewards that the algorithm received (i.e., adherence).

The algorithm used several attributes to predict which factors to select. These included patient baseline characteristics (e.g., age, sex, race/ethnicity, number of medications, concomitant insulin use, self-reported patient activation, education level, employment status, marital status, and therapeutic class), the number of days since each factor had last been sent, and whether the medication had already been taken before the algorithm was run for that day. The algorithm was trained to predict whether or not to include each aspect separately using a “contextual bandit” framework^62–64^. The specific message to be sent was randomly selected from messages matching the required aspects.

The text messages were sent on a daily basis to patients using a third-party SMS platform, including an introductory text and simple reminder text to synchronize their pill bottles if they had not been connected for ≥7 days.

Patients in the control arm received the same introductory and simple reminder text to synchronize their pill bottles if they had not been connected for ≥7 days but otherwise received no intervention.

### Study outcomes

The trial’s primary outcome was medication adherence assessed in the 6 months after randomization using the average daily adherence for each patient (which already averaged across multiple medications)^22^. While other secondary outcomes were measured, we focus on the primary outcome and related analyses in this manuscript.

### Statistical analysis

The overall trial was powered to detect a 10% difference in average adherence over the 6-month follow-up, assuming a SD = 12.5%. We reported key sociodemographic and clinical pre-randomization variables separately for intervention and control using absolute standardized differences (imbalance as a difference >0.1)^65^. Intention-to-treat principles were used for all randomized patients, with a two-sided hypothesis tested at *α* = 0.05. We used SAS 9.4 (Cary, NC) for analyses.

The process and performance of the reinforcement learning algorithm were descriptively examined. The average proportion of patients who received each behavioral factor was estimated and plotted over time for each individual patient. To explore the extent to which adherence was explained by algorithm predictions each day, the adjusted R^2^ based on the algorithm predictions was estimated for each day over the trial. Specifically, we calculated the proportion of variance in daily adherence that was explained by just the five intervention factors. We also explored the most influential features selected by the model when predicting which messages to send for the entire follow-up period; higher scores indicates more influence to the model.

For the primary outcome, we evaluated the effect of the reinforcement learning intervention on adherence using generalized estimating equations with an identity link function and normally distributed errors. These models were adjusted for the block-randomized design, and given imbalances in some important covariates, also controlled for differences in measured baseline characteristics. Some of these imbalances included: more patients in the intervention arm with no more than a high school education (24.1% vs. 16.1%), fewer intervention patients who were married/or partnered (44.8% vs. 54.8%), and more intervention patients taking multiple diabetes medications (31.0% vs. 19.4%). Each of these characteristics have been shown previously to influence adherence^66,67^. Exploratory subgroup analyses were performed according to key demographic/clinical subgroups including age, sex, race/ethnicity, marital status, baseline HbA1c, number of years using oral diabetes medications, baseline self-reported adherence, and number of pill bottle medications. There was no missing data for the primary outcome. Several other sensitivity analyses were also conducted, including omitting the first 14 days for observer effects^44^ and censoring patients in both arms when the pill bottles were not connected for ≥30 days.

Additional exploratory and descriptive analyses of adherence in response to the intervention factors were also conducted for future hypothesis generation. First, the associations between key baseline characteristics and optimal adherence (i.e., adherence value = 1 for the subsequent day) by behavioral factor for intervention patients were explored. To do so, we used generalized estimating equations for each behavioral factor (e.g., positive framing) with a log link and binary-distributed errors with optimal adherence, including all patient baseline characteristics but unadjusted for patient-level clustering due to sample size. Then, we described adherence to different behavioral factors based on the sequence of delivered text messages. Finally, patients were clustered by their average response to different text message factors using *k-*means clustering analysis, using a threshold of ≥25 responses; smaller numbers were replaced by average variable values. Using these clusters, we explored the bivariate association between key baseline patient demographic/clinical characteristics and membership in each group using multinomial logistic regression (Referent: Group 3). Together, these findings may provide a more accurate starting point for future programs.

### Supplementary information

Supplemental Information

## Data Availability

De-identified data necessary to reproduce results reported here are posted on the Harvard Dataverse, an open access repository for research data, at https://dataverse.harvard.edu/. Some additional data, specifically dates such as for example dates of medication use, will be available upon reasonable request and execution of appropriate data use agreements, because dates are Protected Health Information under 45 CFR §164.154(b).

## Code availability

Code necessary to reproduce results reported here are available in the Harvard Dataverse at https://dataverse.harvard.edu/.
