# Supplementary material for: The impact of using reinforcement learning to personalize communication on medication adherence: findings from the REINFORCE trial
Source: NPJ Digit Med. 2024 Feb 19;7:39. doi: 10.1038/s41746-024-01028-5 (PMC10876539; doi:10.1038/s41746-024-01028-5)
Supplement: Supplementary file 1 — Supplemental Information [file 41746_2024_1028_MOESM1_ESM.pdf]

SUPPLEMENTARY INFORMATION

Supplemental Figure 1. Proportion of reinforcement learning patients receiving behavioral factor in text messages over the course of the trial

Note: The individual colors represent whether individual patients included in the intervention arm received that factor that day; the bold line indicates the average across patients for that calendar day.

a. **Receipt of positive framing as a factor**

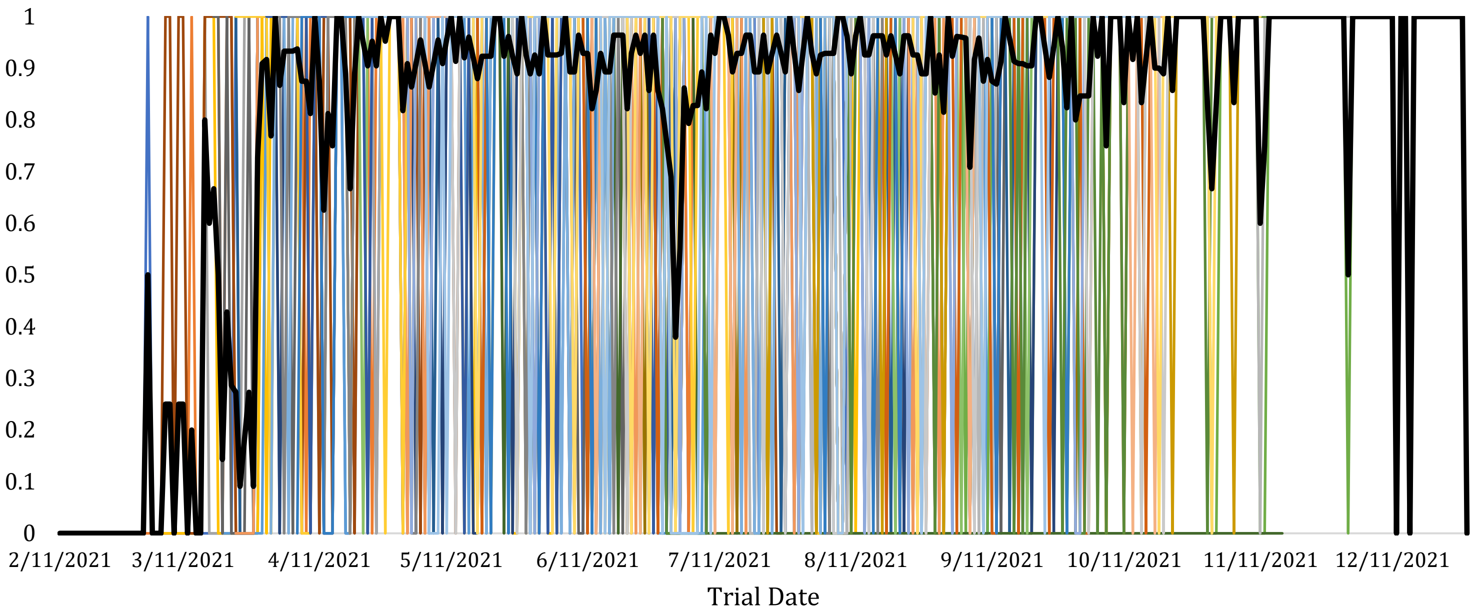

b. **Receipt of negative framing as a factor**

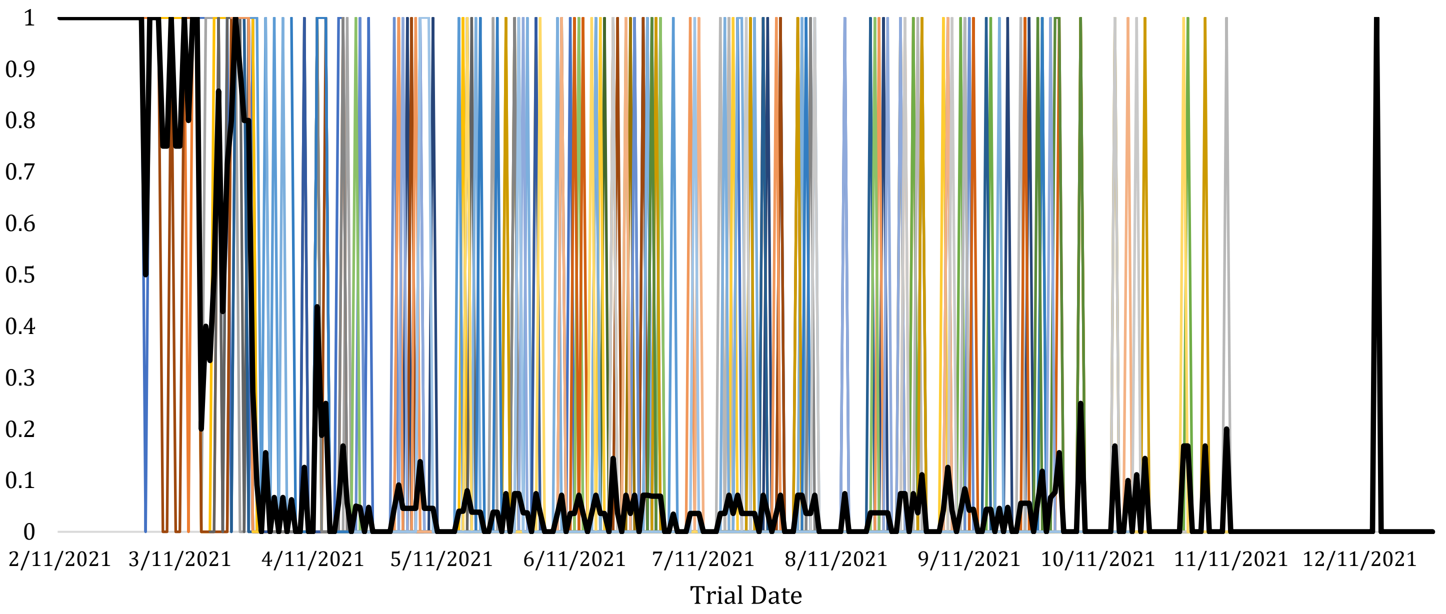

c. Receipt of history (Observed feedback) as a factor

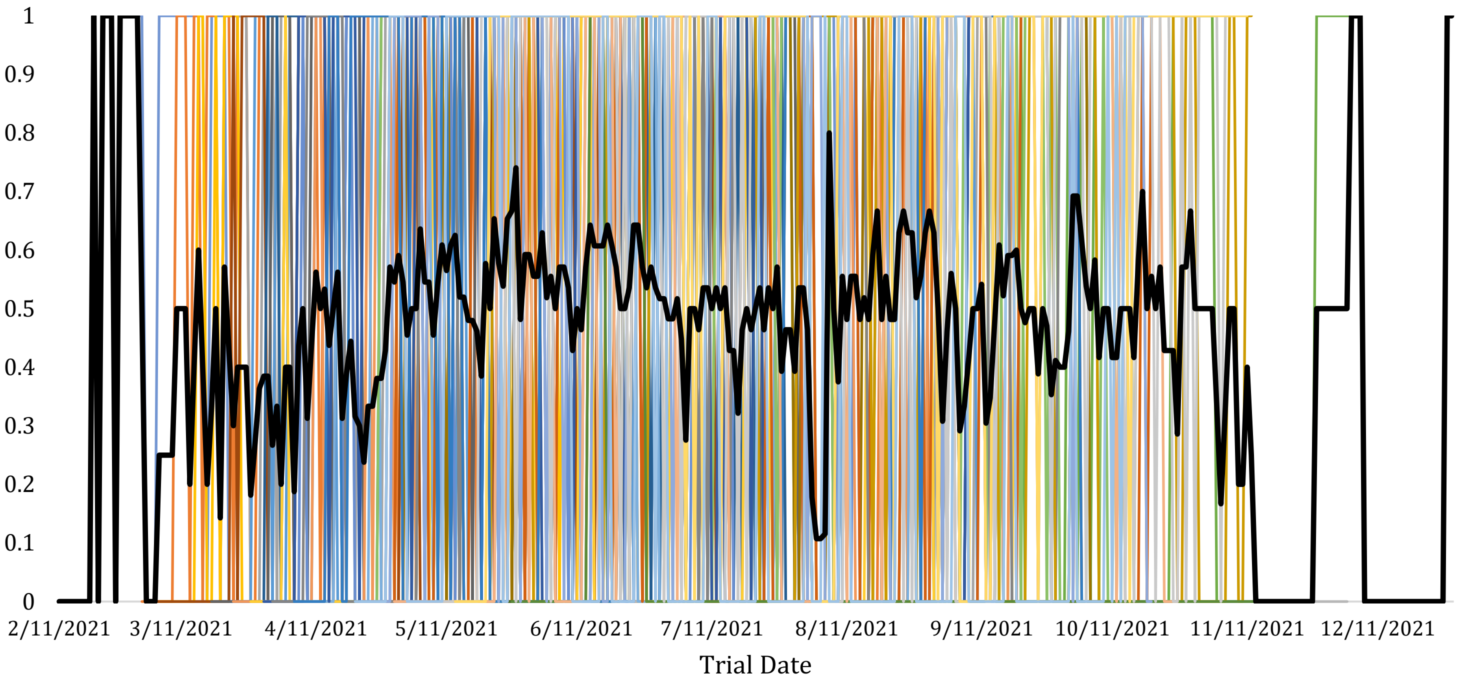

d. Receipt of social reinforcement as a factor

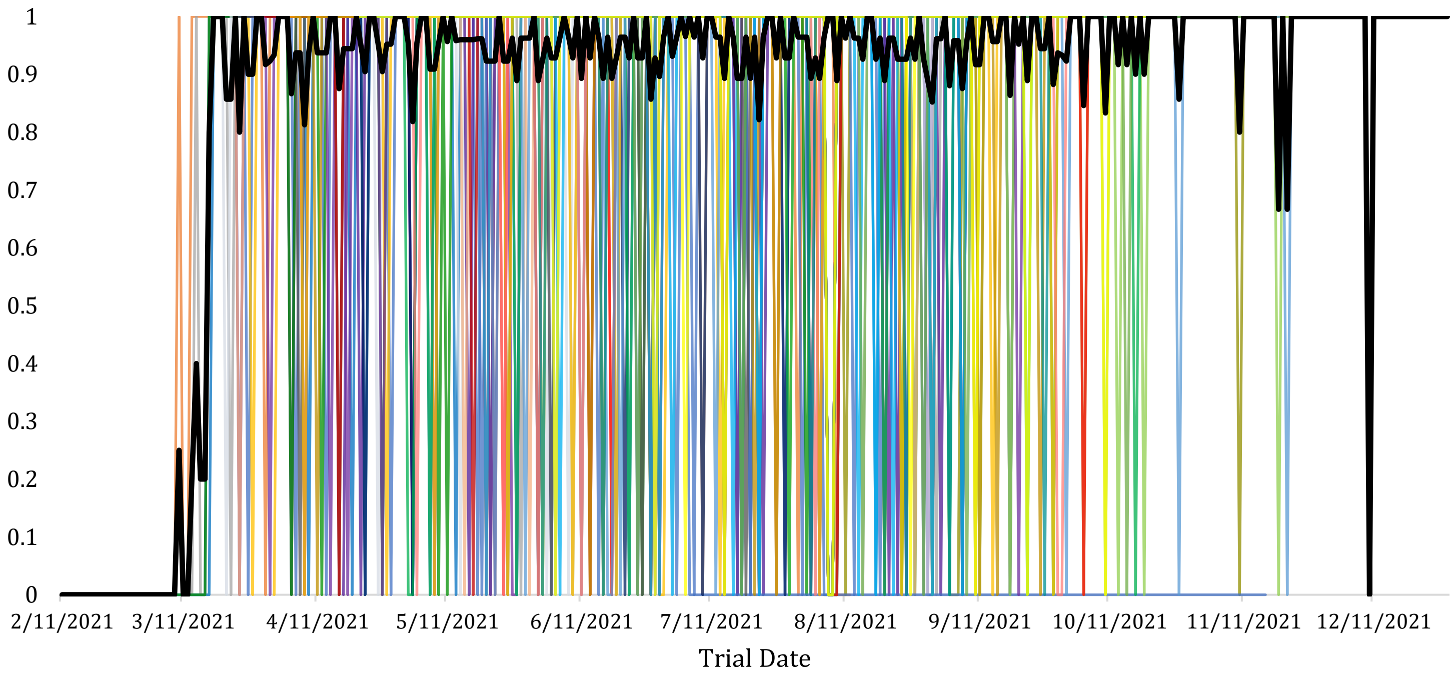

e. Receipt of content as a factor (vs. reminder)

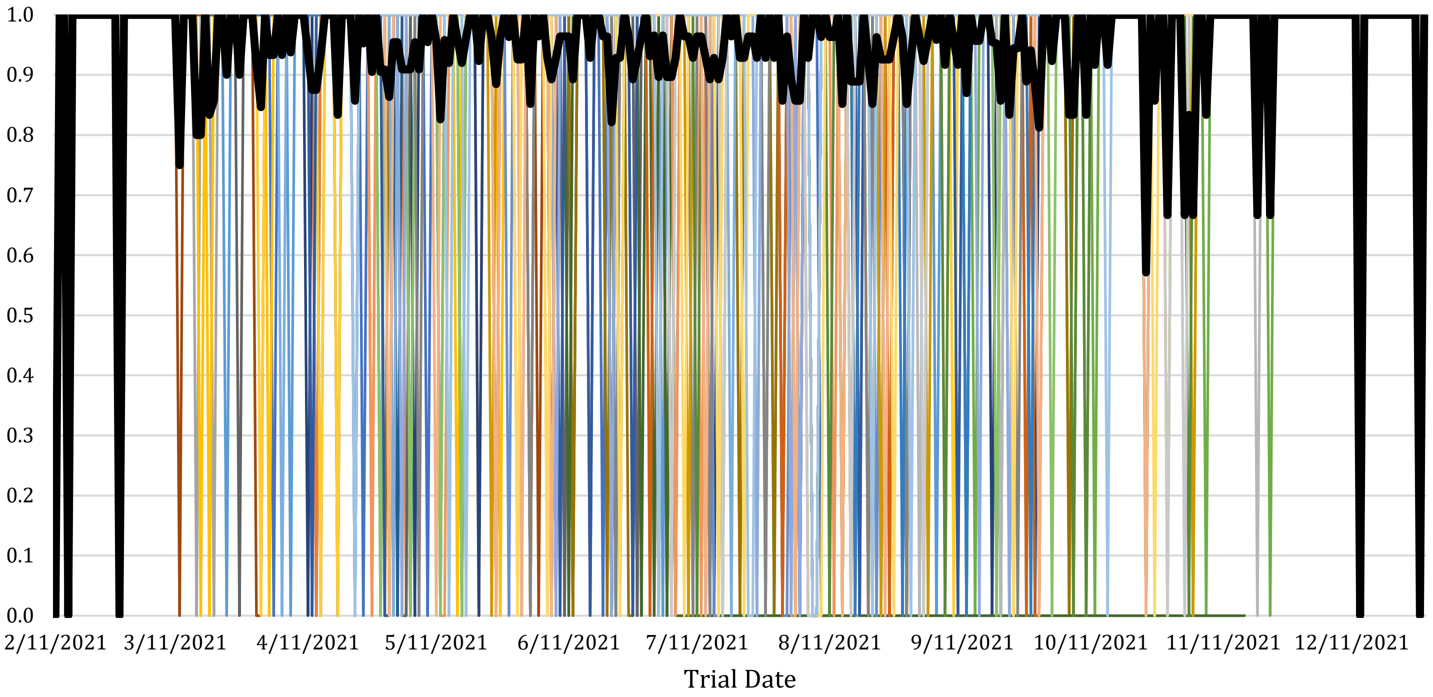

f. Receipt of reflection as a factor (vs. no reflection)

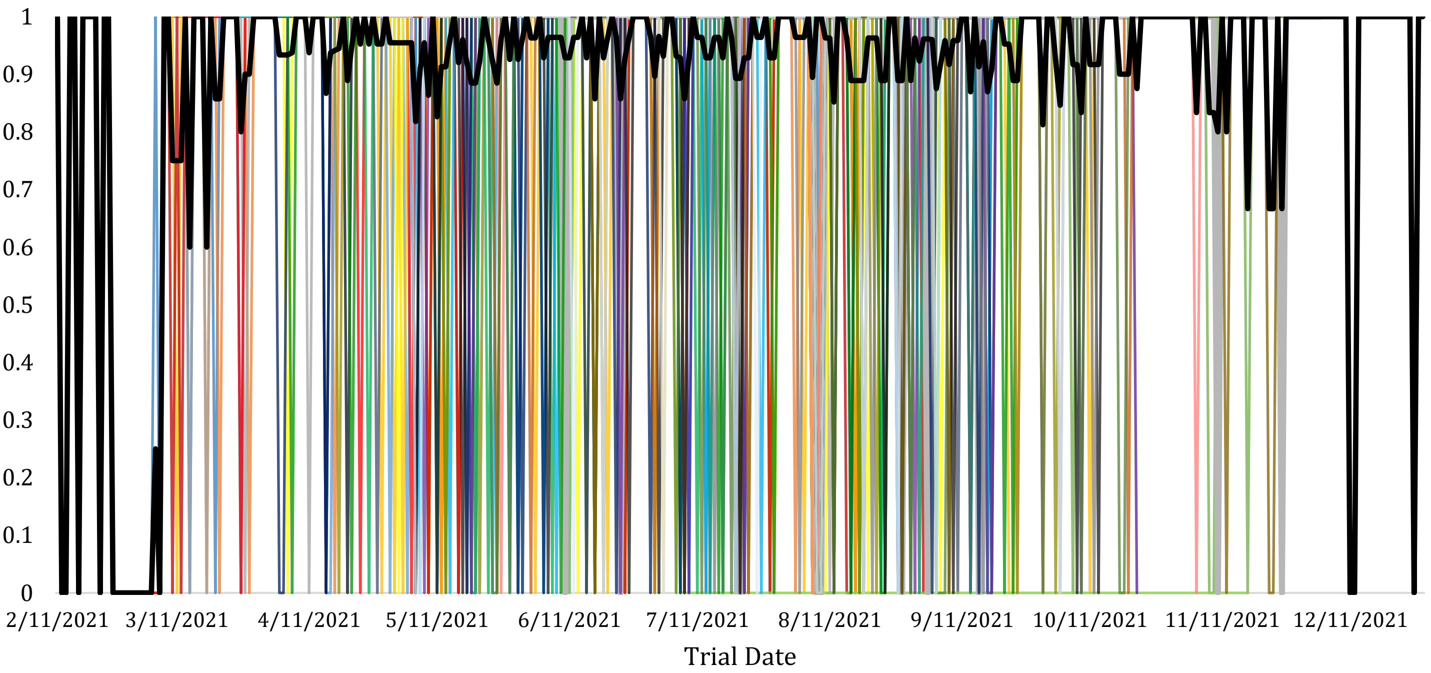

Supplemental Table 1. Sensitivity analyses of the primary outcome

Adherence to medication	Reinforcement learning (N=29) Mean (SD)	Control (N=31) Mean (SD)	Adjusted§ absolute difference, 95%CI
Primary approach	74.3% (30.8%)	67.7% (29.4%)	13.6% (1.7%, 27.1%)*
Omitting first 2 weeks of follow-up	73.5% (31.3%)	66.5% (30.6%)	14.3% (0.5%, 28.2%)*
Censoring after 30 days of pill bottle non-use	75.3% (28.9%)	67.9% (29.0%)	13.6% (0.5%, 26.6%)*

Note: We conducted sensitivity analyses of the primary outcome using generalized estimating equations with an identity link and normally distributed errors, adjusting for the block randomized design and baseline characteristics.

Abbreviations: SD, standard deviation; CI, confidence interval

§Adjusted for baseline variables (listed in Table 1)

*p<0.05
